# Ultraviolet Spectroscopic Detection of Nitrate and Nitrite in Seawater Simultaneously Based on Partial Least Squares

**DOI:** 10.3390/molecules26123685

**Published:** 2021-06-16

**Authors:** Hu Wang, Aobo Ju, Lequan Wang

**Affiliations:** State Key Laboratory of Marine Geology, Tongji University, Shanghai 200092, China; 2031690@tongji.edu.cn (A.J.); 1753079@tongji.edu.cn (L.W.)

**Keywords:** UV spectroscopy, partial least squares, simultaneous determination, nitrate and nitrite, seawater

## Abstract

A direct, reagent-free, ultraviolet spectroscopic method for the simultaneous determination of nitrate (NO_3_^−^), nitrite (NO_2_^−^), and salinity in seawater is presented. The method is based on measuring the absorption spectra of the raw seawater range of 200–300 nm, combined with partial least squares (PLS) regression for resolving the spectral overlapping of NO_3_^−^, NO_2_^−^, and sea salt (or salinity). The interference from chromophoric dissolved organic matter (CDOM) UV absorbance was reduced according to its exponential relationship between 275 and 295 nm. The results of the cross-validation of calibration and the prediction sets were used to select the number of factors (4 for NO_3_^−^, NO_2_^−^, and salinity) and to optimize the wavelength range (215–240 nm) with a 1 nm wavelength interval. The linear relationship between the predicted and the actual values of NO_3_^−^, NO_2_^−^, salinity, and the recovery of spiked water samples suggest that the proposed PLS model can be a valuable alternative method to the wet chemical methods. Due to its simplicity and fast response, the proposed PLS model can be used as an algorithm for building nitrate and nitrite sensors. The comparison study of PLS and a classic least squares (CLS) model shows both PLS and CLS can give satisfactory results for predicting NO_3_^−^ and salinity. However, for NO_2_^−^ in some samples, PLS is superior to CLS, which may be due to the interference from unknown substances not included in the CLS algorithm. The proposed method was applied to the analysis of NO_3_^−^, NO_2_^−^, and salinity in the Changjiang (Yangtze River) estuary water samples and the results are comparable with that determined by the colorimetric Griess assay.

## 1. Introduction

Nitrate (NO_3_^−^) and nitrite (NO_2_^−^) are the essential nutrients for marine phytoplankton growth and play a key role in many biogeochemical cycles [1,2]. NO_3_^−^and NO_2_^−^ concentrations in seawater are also important indicators of water quality. Due to human activities, large amounts of nutrients are discharged into natural waters, thereby destroying the ecological balance and causing the eutrophication of water bodies [3]. Therefore, accurate quantification of NO_3_^−^ and NO_2_^−^ is critical for understanding the dynamics of marine ecosystems. Wet chemical analyses of NO_3_^−^ and NO_2_^−^ in seawater (e.g., the Griess assay) have been previously reviewed in the literature [4,5,6]. These chemical methods require the addition of chemical reagents, and thus, are time-consuming, and waste is generated during measurement.

**Figure 1:**
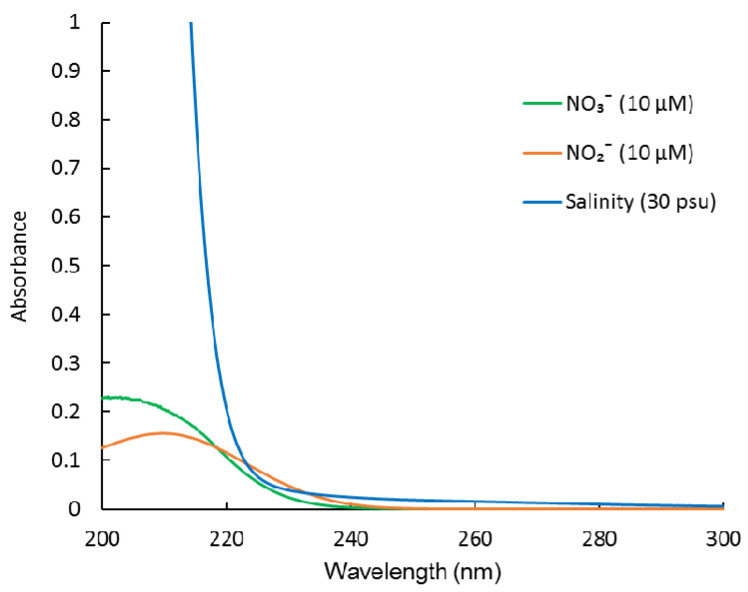
Absorption spectra of NO_3_^−^, NO_2_^−^, and sea salt (salinity).

Ultraviolet (UV) spectroscopy is another well-known method for determining NO_3_^−^, which is based on the strong UV absorption spectrum of NO_3_^−^ [7]. It is a standard method for NO_3_^−^ analysis by the American Public Health Association [8]. The advantages of this method include its simplicity and speed of data acquisition. It avoids the use of any chemical reagents. Therefore, it can easily be developed into an underwater sensor for long-term monitoring. However, this method is susceptible to interference from high concentrations of Cl^−^ and Br^−^ (or sea salt, salinity) in seawater, which have strong UV absorbance in the NO_3_^−^ absorption range [9]. Previously, multi-wavelength measurement and classic least square (CLS) regression were used to separate the overlapping spectra and measure NO_3_^−^ [9,10,11,12]. However, it did not take NO_2_^−^ into account. Although NO_2_^−^ is the least abundant of the major inorganic nitrogen ions (NH_4_^+^, NO_3_^−^, and NO_2_^−^) [13], it can accumulate at concentrations up to 10 μM in low-oxygen estuary and coastal waters, oxygen-deficient zones, and upwelling regions [14,15,16,17,18]. In these areas, NO_2_^−^ may influence the NO_3_^−^ measurement considering NO_2_^−^ has a similar UV absorption spectrum to that of NO_3_^−^ (Figure 1). To date, there is no report on simultaneous determination of NO_3_^−^ and NO_2_^−^ in seawater using UV spectroscopy. Langergraber et al. (2003) and Rieger et al. (2004, 2008) used a submersible UV/VIS spectrometer combined with partial least squares (PLS) regressions to monitor NO_3_^−^, NO_2_^−^, and even chemical oxygen demand, in the effluent of municipal wastewater treatment plants [19,20,21]. However, this method cannot be applied to analyze NO_3_^−^ and NO_2_^−^ in seawater. The reason for this is that they do not eliminate the interference from sea salt and chromophoric dissolved organic matter (CDOM) in seawater.

In this study, we aimed to (1) develop a direct, reagent-free, ultraviolet spectroscopic method to determine NO_3_^−^, NO_2_^−^, and salinity simultaneously based on the PLS model: (2) select an appropriate number of factors and optimal wavelength range for the PLS model; (3) evaluate the performance of the proposed method; (4) compare the results of the PLS and CLS models; (5) apply the proposed method to estuarine water samples.

## 2. Theory

### 2.1. PLS Regressions

PLS is a kind of multivariate calibration method based on factor analysis. It is a combination of principal component analysis, multiple linear regression analysis, and canonical correlation analysis. The theoretical basis for PLS regression can be found in several references [22,23,24,25,26]. PLS establishes a quantitative relationship (Equations (1) and (2)) between an n × m matrix (X) of independent variables (absorbance at each wavelength, in this case) and an n × k matrix (Y) of the predicted values of the variables (NO_3_^−^, NO_2_^−^, and salinity, in this case). The PLS model can be written as follows

X = TP^T^ + E (1)

Y = UQ^T^ + F (2)

where P and Q are the loading matrices of X and Y, which give information about weights for each predictor in X when calculating latent variables (factors); E and F are the matrices of X and Y residuals (both with the same dimensions as the original absorbance and concentration matrices, respectively); T and U represent the score matrix for X and Y, which can summarize X and predict Y with small errors in E and F. The decompositions of X and Y are made to maximize the covariance between T and U. The PLS model is built using a calibration or training set of samples that have known property values. Following the establishment of a satisfactory model, the property values in a prediction set of samples can be predicted. Here, the experimental spectra (matrix X) of single and mix standards, with known concentrations NO_3_^−^, NO_2_^−^, and salinity (matrix Y), were used as the calibration set. 

PLS calibration of a multi-component system can be performed in two different ways, PLS1 and PLS2. In PLS1, a separate set of scores and loading vectors is tuned and calculated for each variable (NO_3_^−^, NO_2_^−^, and salinity). In PLS2, several variables (NO_3_^−^, NO_2_^−^, and salinity) are fitted simultaneously, and there is one common set of factors for NO_3_^−^, NO_2_^−^, and salinity [25,27]. Therefore, PLS1 should give more accurate predictions than PLS2, especially when one of the variables is influenced by a number of factors different to other variables in the mixture [26,28,29]. However, PLS2 can simplify the procedure and allows for simultaneous graphical inspection. Thus, PLS2 is faster to use than PLS1. However, it should be noted that PLS2 usually performs equally well or worse than PLS1 if there is a weak or no correlation between response variables [30,31]. In the present study, the results from PLS1 and PLS2 models are compared.

### 2.2. Interference from CDOM

CDOM comprises a significant fraction of the DOM pool in natural waters (~10–90%) [32]. It has strong absorption in the UV range [33,34]. Thus, the interference with NO_3_^−^ and NO_2_^−^ from CDOM is of particular concern. CDOM is a mixture of many organic compounds that differ spatially and temporally due to their origin. The spectral shapes of CDOM vary with the compositions of CDOM. It is difficult to link optical absorbance directly to CDOM concentrations or compositions [35,36]. Therefore, CDOM cannot be added to the predictor variable list for the model building. Many previous studies have suggested that the UV absorption spectrum of CDOM in seawater fits an exponential function with wavelengths [36,37,38,39], which can be given in Equation (3)

(3) $$A_{\mathrm{CDOM}}\left(\lambda \right)=A_{\mathrm{CDOM}}\left(\lambda_{0}\right)e^{S\left(\lambda_{0}-\lambda \right)}+k$$

where λ is the wavelength (nm), λ_0_ is a reference wavelength (nm); A_CDOM_(λ) and A_CDOM_($\lambda_{0}$) are the CDOM absorbance at the wavelength of λ and λ_0_; k is a background constant (m^−1^), which accounts for light scattering in the cuvette and drift of the instrument. S is the spectral slope (nm^−1^) that describes the approximate exponential rate of decrease in absorption with increasing wavelengths. In Equation (3), S and a are used to define differences among different samples. Usually, S is calculated over a broad wavelength range (e.g., 275–295, 350–400, and 300–600 nm) [36,38,39]. Several previous studies used a quadratic function [9,40] or linear function [41,42] to fit, approximately, the CDOM spectra. Given the comparatively high concentrations of CDOM in estuarine and coastal waters, in this study, we used an exponential function to fit the absorption spectrum of CDOM between 275 to 295 nm. The wavelength 300 nm was chosen as the reference wavelength (λ_0_). Then this function was applied to the wavelength from 200 to 240 nm. Thus, the CDOM absorbance can be subtracted from the raw spectra in the developed PLS model. 

### 2.3. CLS Regression

CLS is the simplest and most widely used technique for solving overdetermined systems. In its most important application—data fitting—it finds a hyperplane through a set of data points while minimizing the sum of squared errors [43,44]. For comparison with PLS, we also used CLS regression to fit the absorbance spectra of seawater samples and obtain NO_3_^−^ and NO_2_^−^ concentrations according to Equation (4)

(4) $$A_{\lambda }=b\left(\varepsilon_{{\mathrm{NO}}_{3}{}^{-}}\times C_{{\mathrm{NO}}_{3}{}^{-}}+\varepsilon_{{\mathrm{NO}}_{2}{}^{-}}\times C_{{\mathrm{NO}}_{2}{}^{-}}+\varepsilon_{\mathrm{salinity}}\times \mathrm{salinity}\right)+A_{\mathrm{CDOM}}$$

where b is the pathlength (cm) of the optical cell, ε is the absorption coefficient of the subscripted species (L mol^−1^ m^−1^ for NO_3_^−^ and NO_2_^−^, PSU^−1^ m^−1^ for salinity), C is the concentration of the subscripted species. Each ε value can be obtained by measuring the absorption in standard solutions of each chemical species. The concentrations of NO_3_^−^, NO_2_^−^, salinity, and all the CDOM coefficients (Equation (4)), were fitted together.

## 3. Experimental

### 3.1. Reagents

All chemicals were of analytical reagent grade and supplied by the Sigma-Aldrich Company (Shanghai, China). The standard solutions of NO_3_^−^ and NO_2_^−^ were freshly prepared from NaNO_3_, NaNO_2_, and deionized water (Milli-Q water, 18.2 MΩ) before use. 

### 3.2. Apparatus and Software

A UV-Vis spectrophotometer (Specord plus 210, Analytik Jena AG, Germany) was used to collect absorbance data from 200 to 300 nm. Due to the comparatively low concentrations and absorbance of NO_2_^−^, all the samples were measured in a 3.0 cm quartz cuvette. Milli-Q water was used as the reference. The spectral resolution was set as 1 nm. A higher resolution (e.g., 0.2–0.8 nm) yields similar results. All data-processing scripts, including PLS1, PLS2 (see the Supplementary Materials), and CLS regressions, were written in MATLAB for Windows (Mathworks, version 2019b).

### 3.3. Model Validation

The evaluation of the modelling error was obtained from the analysis of the predicted vs. actual concentration plots, being the root mean square error of the prediction data (RMSEP) which provides information about the fit of the model to the calibration data, the correlation coefficient (R^2^) between predicted and actual concentration values of the prediction set, and the relative percentage error in concentration prediction (RE). These definitions are as follows (Equations (5)–(7))

(5) $$\mathrm{RMSEP}=\sqrt{\frac{1}{N}\overset{N}{\underset{i=1}{\sum }}{\left(C_{i}-{\hat{C}}_{i}\right)}^{2}}$$

(6) $$R^{2}=\frac{\left[\sum_{1}^{N}(C_{i}-\bar{C_{i}}\right)\left({\hat{C}}_{i}-\bar{{\hat{C}}_{i}}\right){]}^{2}}{\sum_{1}^{N}{\left(C_{i}-\bar{C_{i}}\right)}^{2}\sum_{1}^{N}{\left({\hat{C}}_{i}-\bar{{\hat{C}}_{i}}\right)}^{2}}$$

(7) $$\mathrm{RE}=\frac{1}{N}\overset{N}{\underset{i=1}{\sum }}\frac{{\hat{C}}_{i}-C_{i}}{C_{i}}$$

where C_i_, and ${\hat{C}}_{i}$ are the real and predicted concentration in the ith sample, $\bar{C_{i}}$ and $\bar{{\hat{C}}_{i}}$ are the mean of the real and the predicted concentrations of all the samples in the predicted sets. N is the number of samples in the prediction set.

### 3.4. The Calibration and Prediction Sample Sets

The samples for the calibration and prediction sets were prepared using seawater samples collected in the Changjiang estuary with known concentrations of NO_3_^−^, NO_2_^−^, and salinity spiked with NO_3_^−^ and NO_2_^−^ standard solutions. An experimental design was used to construct the calibration set to provide a good prediction. As shown in Table 1, 34 samples were selected as the training and the prediction set, which included one-, two- and three-components of NO_3_^−^, NO_2_^−^, and salinity with various concentrations. For the prediction set of 20 samples, their compositions were randomly designed within similar ranges of NO_3_^−^, NO_2_^−^, and salinity in the training set.

## 4. Results and Discussion

### 4.1. Selection of the Optimal Number of Factors 

The number of factors, or latent variables, is an important parameter governing the performance of the PLS model. The introduction of an unnecessary number of factors may result in the overfitting of the calibration curve. To select the number of latent variables in PLS regression, a cross-validation procedure of leaving out one sample at a time was employed for PLS in order to model the compositions without overfitting the data [22,26,45]. From the set of 34 calibration spectra, the PLS calibration was performed on 33 spectra. Using this calibration, the concentration of the compounds in the sample left out was predicted. This process was repeated 34 times until each calibration sample had been left out once during the calibration process. The concentration predicted for each sample was then compared with its known concentration. The sum of the squared concentration prediction errors for all calibration samples (prediction error sum of squares (PRESS)) was used to determine how well a particular PLS model fitted the concentration data. This is defined in Equation (7).

One reasonable choice for the optimum number of factors (h) would be the number that yielded a minimum PRESS value. However, in many cases, the minimum PRESS value resulted in the overfitting of the data, given that it is based on a finite number of samples and thus subject to error [22,43]. A frequently used methodology to determine h is based on both the value of PRESS and a Q^2^ threshold, defined as follows (Equations (8)–(10)) [46,47].

(8) $${\mathrm{PRESS}}_{h}=\overset{n}{\underset{i=1}{\sum }}{\left(C_{i}-{\hat{C}}_{h\left(-i\right)}\right)}^{2}$$

(9) $${\mathrm{RESS}}_{h-1}=\overset{n}{\underset{i=1}{\sum }}{\left(C_{i}-{\hat{C}}_{\left(h-1\right)i}\right)}^{2}$$

(10) $$Q_{h}^{2}=1-\frac{{\mathrm{PRESS}}_{h}}{{\mathrm{RESS}}_{\left(h-1\right)}}$$

where PRESS_h_ is the predictive residual error sum of squares when the number of components is equal to h. RESS_h−1_ is the residual sum of squares when the number of components is h−1. C_i_ is the real concentration of the analyte and ${\hat{C}}_{h(-i)}$ is the fitting concentration of the analyte in the ith sample computed by the PLS regression after deleting the ith sample and using h factors. ${\hat{C}}_{(h-1)i}$ is the fitting concentration of the analyte in the ith sample computed by the PLS after using all the sample points and h−1 factors.

The factor h is considered significant (*p* ≤ 0.05) for the prediction [43,44].

(11) $$\sqrt{{\mathrm{PRESS}}_{h}}\leq 0.95\sqrt{{\mathrm{RESS}}_{h-1}}⇋Q_{h}^{2}\geq 0.0975$$

When Q_h_^2^ is less than 0.0975, adding another factor does not improve model precision. For the PLS1 model, a cross-validation procedure was run three times for NO_3_^−^, NO_2_^−^, and salinity separately; thus, the factors were also calculated for NO_3_^−^, NO_2_^−^, and salinity separately. For the PLS2 model, the cross-validation was run only once and the number of factors were calculated only once for NO_3_^−^, NO_2_^−^, and salinity simultaneously. Take the wavelength range of 215–240 nm as an example; the PRESS and Q_h_^2^ of PLS1 and PLS2 models are shown in Table 2. It can be seen that both the PLS1 and PLS2 models give the same factors of 4 for NO_3_^−^, NO_2_^−^, and salinity. The cumulative contribution rates for 4 factors reached 99.99%.

### 4.2. Wavelength Selection

The wavelength selection is carried out to choose a subset of spectral channels with which the established calibration model can give the minimum errors of the prediction. The optimal wavelength selection offers two clear benefits. Firstly, it has been shown that the inclusion of uninformative wavelengths in the training process negatively affects the accuracy of predictions and model interpretability [48,49]. Secondly, from a more practical point of view, the identification of a few wavelengths, or regions of the optical spectrum, that contain information about chemical species, significantly reduces the time and cost associated with their measurement and enables the development of portable and high-speed optical sensors.

**Figure 2:**
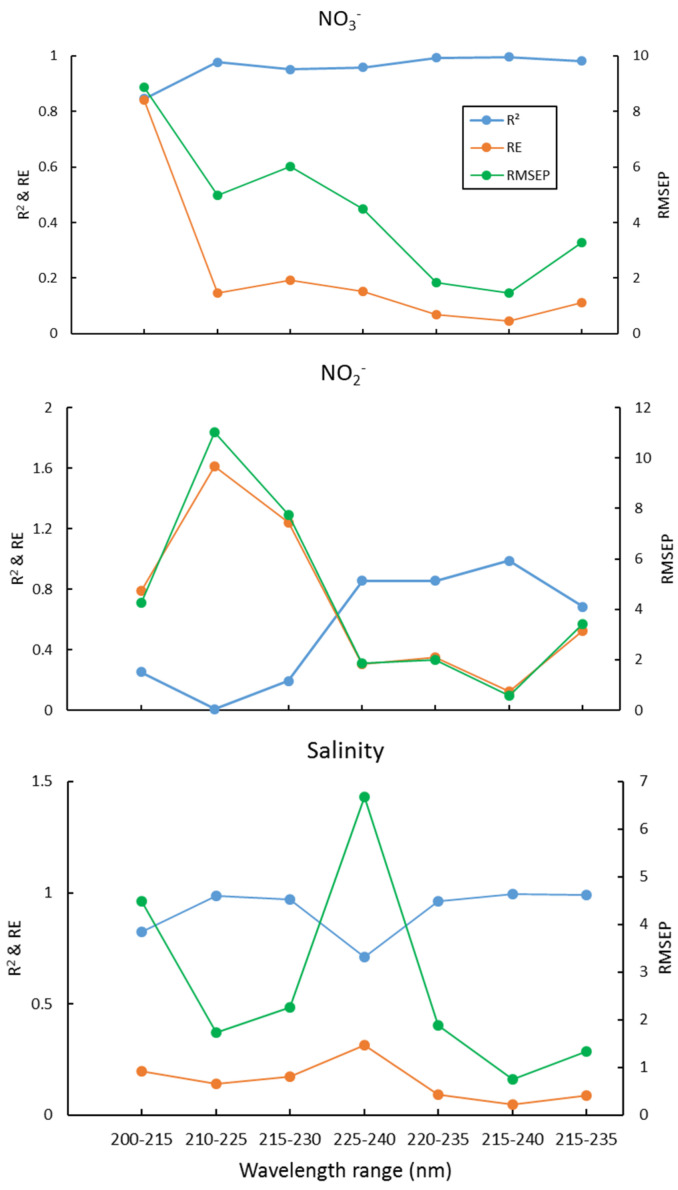
R^2^, RE, and RMSEP of NO_3_^−^, NO_2_^−^, and salinity for different wavelength ranges.

**Figure 3:**
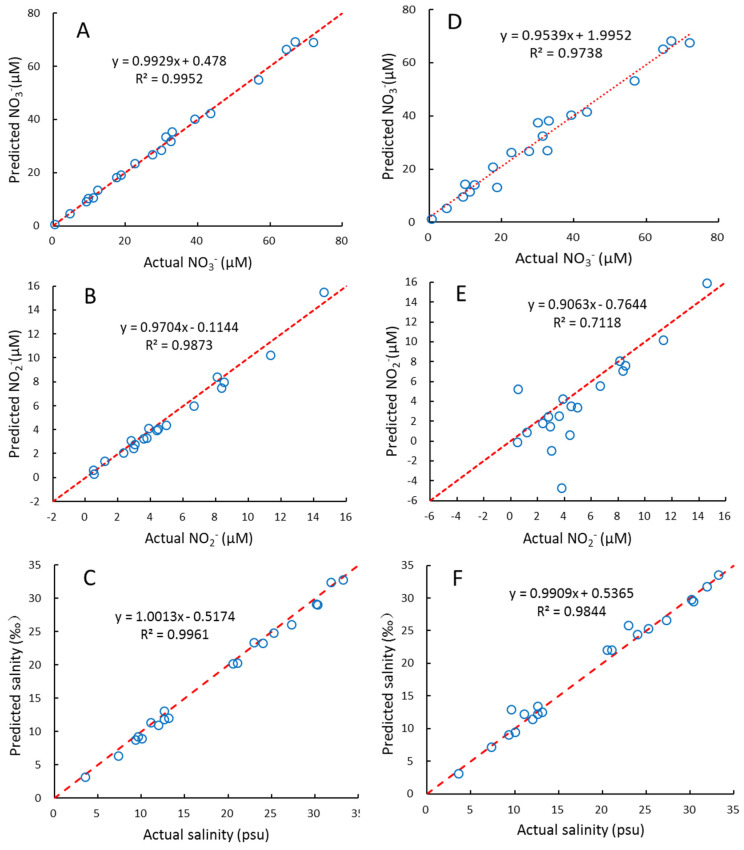
The relationships between the actual and predicted NO_3_^−^, NO_2_^−^ and salinity of samples in prediction set using PLS2 model (**A**–**C**) and CLS model (**D**–**F**). Red dash lines indicate ratio of actual: predicted value = 1:1.

We used interval partial least squares (iPLS) to optimize the wavelength selection proposed by Norgaard et al. (2000) [50], which is to split the spectrum into different intervals and treat each interval as a variable, then the RMSEP, R^2^, and RE for each interval was calculated. The interval with maximal R^2^, and minimum RMSEP and RE, was chosen as the optimal wavelength interval. Here, the 200–300nm wavelength is equally divided into equal subintervals of 16 nm, 200–215 nm, 210–225, 220–235, …, 285–300 nm. Then the wavelength range with the lowest RMSEP value was chosen for further optimization using one-sided symmetrical optimization. The results of the PLS2 for several wavelength ranges are shown in Figure 2. This suggests that the optimal wavelength interval is 215–240 nm, which gives the maximal R^2^, and minimal RE and RMSEP, for NO_3_^−^, NO_2_^−^, and salinity simultaneously. The plots of these predicted concentrations versus actual concentrations using the PLS2 model are shown in Figure 3. As can be seen, the predicted NO_3_^−^, NO_2_^−^, and salinity predicted are linearly correlated with the actual values, and all the correlation coefficients are > 0.98 (Figure 3). As both the PLS1 and PLS2 models have 4 factors, the results of the PLS1 are the same as the PLS2. For reducing the complexity and computation time of the model, we recommend using the PLS2 model and wavelength of 215–240 nm for calibration and prediction.

### 4.3. Comparison of PLS2 and CLS Regressions

For comparison, we also built a CLS model to fit the NO_3_^−^, NO_2_^−^, and salinity based on Equation (4). The results obtained for the samples of the prediction set are shown in Figure 3. For NO_3_^−^ and salinity, the results of CLS are also satisfactory. It is similar to previous studies [9,40,42], in which different CLS algorithms were used to fit NO_3_^−^ concentrations and salinity in seawater. However, for several samples with low NO_2_^−^ concentrations, the CLS model is less predictive than the PLS2 model. The reason for this may be that NO_2_^−^ concentrations in seawater are significantly lower than NO_3_^−^, and thus, NO_2_^−^ is more susceptible to the interference of sea salt, CDOM, hydrogen sulfide, and other unknown substances. Instead, PLS regression, as an indirect chemometric method, can lead to robust results even if not all the constituents are known [22,23].

### 4.4. Evaluation of the PLS2 Model

From the experimental data illustrated in Table 1, NO_3_^−^, NO_2_^−^, and salinity within the range of 0–85.62 μM, 0–20.12 μM, and 0–33.90 psu can be determined accurately by the PLS model. When NO_3_^−^ concentration is higher than 100 μM, we suggest using a cuvette with a 1.0 or 2.0 cm pathlength instead of 3.0 cm in case the absorbance tends to saturate. To further evaluate the accuracy of the PLS2 model, recovery studies were carried out on seawater samples, to which known amounts of NO_3_^−^ and NO_2_^−^ were added (Table 3). The percentage recovery for spiked samples ranged between 80 and 110%. The comparatively higher deviations from spiked concentrations were obtained from samples with low NO_3_^−^ or NO_2_^−^ concentrations. The detection limits of NO_3_^−^ and NO_2_^−^ in seawater were calculated as three times the standard deviation of 10 replicate analyses of a low-nutrient (surface) seawater. The standard deviation of the measurements was 0.07 and 0.10 μM for NO_3_^−^ and NO_2_^−^, which gives NO_3_^−^ and NO_2_^−^ detection limits of 0.21 and 0.30 μM, respectively. The relative standard deviations for 10 repetitive analyses (*n* = 10) of a standard solution (3.71 μM NO_3_^−^ + 1.48 μM NO_2_^−^) were 3.16% and 7.42%, and another solution of 20.75 μM NO_3_^−^ and 6.28 μM NO_2_^−^ gave the relative standard deviation of 0.61% and 2.39%. Hence, the proposed method is quite precise for the quantitative determination of NO_3_^−^ and NO_2_^−^ in seawater, although the detection limits are comparatively higher than that of most colorimetric methods based on the Griess reaction [4,5,6]. Most importantly, it offers a simple, fast, and reagent-free method for the simultaneous determination of NO_3_^−^ and NO_2_^−^.

### 4.5. Application and Comparison of the Predicted Results with Conventional Wet-Chemical Analyses

To evaluate the analytical applicability of the proposed PLS2 model, it was applied to the simultaneous determination of NO_3_^−^, NO_2_^−^, and salinity in water samples collected from the Changjiang estuary. These samples were filtered using 0.2μm polycarbonate filters to eliminate the interference from turbidity. For comparison, the NO_3_^−^ and NO_2_^−^ concentrations were also measured by conventional wet-chemical analyses (colorimetric Griess assay). The results are shown in Table 4, which suggests the good agreement of both methods.

It should be noted that the in situ UV absorption spectrum, which is obtained at different temperatures, should be corrected according to the temperature dependence of bromide or sea salt, as suggested by Sakamoto et al. (2009, 2017) [41,51]. However, here, we measured the UV absorption spectra in a laboratory at room temperature (~25 °C). Therefore, there is no need to perform the temperature and pressure correction.

## 5. Conclusions

A direct, reagent-free, ultraviolet spectroscopic was introduced for the simultaneous determination of NO_3_^−^, NO_2_^−^, and salinity in seawater. A PLS model was built for the resolution of the high overlapping spectra. This method has detection limits of 0.21 and 0.3 μM for NO_3_^−^ and NO_2_^−^. It can be successfully used to determine NO_3_^−^, NO_2_^−^, and salinity, especially in estuarine and coastal waters with varying CDOM characteristics and different salinities. The simplicity, precision, and fast response time suggest that the proposed PLS model can be a valuable and cheap alternative to other chemical methods and can be used to build NO_3_^−^ and NO_2_^−^ sensors for seawater.

## Figures and Tables

**Table 1:** Composition of the calibration and prediction samples.

Calibration Set Samples							
No.	NO_3_^−^ (μM)	NO_2_^−^ (μM)	Salinity (psu)	No.	NO_3_^−^ (μM)	NO_2_^−^ (μM)	Salinity (psu)
1	0.00	0.00	0.00	18	0.97	0.00	33.24
2	0.50	0.00	0.00	19	0.00	9.15	12.33
3	1.98	0.00	0.00	20	0.00	1.93	26.08
4	9.92	0.00	0.00	21	0.00	0.50	33.56
5	49.61	0.00	0.00	22	4.75	0.96	32.44
6	0.00	0.50	0.00	23	20.89	5.30	26.76
7	0.00	2.01	0.00	24	0.95	0.97	26.08
8	0.00	10.06	0.00	25	5.25	1.06	17.94
9	0.00	20.12	0.00	26	11.67	2.96	19.94
10	0.00	0.00	6.78	27	8.27	1.05	14.13
11	0.00	0.00	16.95	28	25.44	10.32	13.91
12	0.00	0.00	27.12	29	4.75	0.96	8.11
13	0.00	0.00	33.90	30	11.81	2.40	10.09
14	4.75	0.96	0.00	31	20.89	5.30	8.92
15	20.89	5.30	0.00	32	52.22	10.59	8.92
16	55.12	0.00	15.07	33	85.62	2.22	1.51
17	9.45	0.00	25.83	34	26.85	6.69	8.86
**Prediction Set Samples**							
1	9.23	1.17	12.61	11	67.18	11.04	7.04
2	0.49	0.49	33.24	12	71.87	4.86	20.69
3	4.61	2.34	25.23	13	66.87	5.07	20.54
4	11.02	2.79	30.13	14	18.66	0.52	9.58
5	27.54	7.95	9.32	15	32.56	15.97	3.56
6	34.20	6.65	12.59	16	12.35	8.68	23.98
7	43.52	8.50	10.06	17	29.95	3.76	32
8	39.12	14.6	11.25	18	56.71	3.56	12.54
9	23.24	4.26	29.32	19	18.11	2.83	23.65
10	9.68	4.05	11.23	20	35.9	2.91	26.57

**Table 2:** The Q_h_^2^ values of cross-validation with the number of factors.

Factors	1	2	3	4	5	6	7
**PLS1**							
PRESS-NO_3_^−^	1962	1391	327.6	2.39	2.51	2.53	3.96
Q_h_^2^-NO_3_^−^	-	0.17	0.77	0.99	−0.36	−0.75	−2.13
Cumulative Contribution Rate -NO_3_^−^ (%)	91.34	99.50	99.95	99.99			
PRESS-NO_2_^−^	614.3	504.6	191.7	7.24	8.11	9.13	5.81
Q_h_^2^-NO_2_^−^	-	0.02	0.48	0.95	−1.30	−2.59	−2.35
Cumulative Contribution Rate -NO_2_^−^ (%)	90.52	99.60	99.95	99.99			
PRESS-Salinity	3552	951.9	61.34	9.64	11.67	12.01	11.67
Q_h_^2^-Salinity	-	0.72	0.92	0.77	−0.53	−0.78	−1.00
Cumulative Contribution Rate -Salinity (%)	76.89	99.62	99.96	99.99			
**PLS2**							
PRESS	7432	3651	543.4	19.27	21.89	23.17	18.79
Q_h_^2^	-	0.47	0.80	0.95	−0.68	−0.95	−0.91
Cumulative Contribution Rate (%)	91.30	99.62	99.95	99.99			

**Table 3:** Recoveries of NO_3_^−^ and NO_2_^−^ in spiked seawater samples.

Spiked (μM)		Found (μM)		Recovery (%)	
NO_3_^−^	NO_2_^−^	NO_3_^−^	NO_2_^−^	NO_3_^−^	NO_2_^−^
3.53	2.10	3.22	1.92	109.74	109.36
10.95	14.20	11.23	13.83	97.50	102.66
19.46	1.94	18.83	2.43	103.36	79.97
28.15	6.28	29.26	6.35	96.22	98.85
28.78	3.83	28.18	4.27	102.11	89.75
37.82	5.66	37.17	5.98	101.76	94.69
46.62	7.44	45.90	7.45	101.57	99.93
55.17	9.18	54.54	9.26	101.16	99.13

**Table 4:** The predicted values of NO_3_^−^, NO_2_^−^, and salinity (average ± 1 standard deviation of three replicate analyses) using the PLS2 model in seawater samples of the Changjiang Estuary.

Sample	NO_3_^−^ (μM)	NO_2_^−^ (μM)	Salinity (psu)
1	11.96 ± 0.13 (10.55 ± 0.54)	4.63 ± 0.13 (3.59 ± 0.12)	23.53 ± 0.04
2	75.73 ± 0.32 (74.68 ± 1.26)	3.73 ± 0.17 (3.14 ± 0.09)	17.07 ± 0.37
3	51.60 ± 0.26 (52.19 ± 1.77)	2.47 ± 0.19 (1.57 ± 0.21)	21.30 ± 0.43
4	33.36 ± 0.08 (31.85 ± 0.89)	7.41 ± 0.08 (6.53 ± 1.12)	23.83 ± 0.01
5	19.36 ± 0.12 (20.73 ± 0.63)	1.27 ± 0.11 (0.67 ± 0.09)	35.29 ± 0.21
6	21.27 ± 0.11 (20.06 ± 0.42)	0.11 ± 0.16 (0.26 ± 0.04)	29.13 ± 0.21
7	73.67 ± 0.09 (72.12 ± 1.02)	0.47 ± 0.13 (1.03 ± 0.13)	3.66 ± 0.04
8	14.18 ± 0.14 (12.53 ± 0.56)	−0.35 ± 0.11 (0.49 ± 0.08)	31.99 ± 0.21
9	68.88 ± 0.28 (67.18 ± 1.55)	5.92 ± 0.22 (4.25 ± 0.06)	20.56 ± 0.16

Notes: NO_3_^−^ and NO_2_^−^ concentrations shown in brackets were determined by the Griess assay.

## Data Availability

The data presented in this study are available on request from the corresponding author.

## Supplementary Materials

All data-processing scripts for this article can be found online.

## Sample Availability

Samples of the compounds are not available from the authors.
